# Clinical Profile and Visual Outcome of Ocular Bartonellosis in Malaysia

**DOI:** 10.1155/2017/7946123

**Published:** 2017-02-07

**Authors:** Chai Lee Tan, Lai Chan Fhun, Evelyn Li Min Tai, Nor Hasnida Abdul Gani, Julieana Muhammed, Tengku Norina Tuan Jaafar, Liza Sharmini Ahmad Tajudin, Wan-Hazabbah Wan Hitam

**Affiliations:** ^1^Department of Ophthalmology, School of Medical Sciences, Universiti Sains Malaysia, Health Campus, 16150 Kubang Kerian, Kelantan, Malaysia; ^2^Department of Ophthalmology, Hospital Raja Permaisuri Bainun, 30990 Ipoh, Perak, Malaysia; ^3^Department of Ophthalmology, Hospital Raja Perempuan Zainab 2, 15586 Kota Bharu, Kelantan, Malaysia

## Abstract

*Background.* Ocular bartonellosis can present in various ways, with variable visual outcome. There is limited data on ocular bartonellosis in Malaysia.* Objective.* We aim to describe the clinical presentation and visual outcome of ocular bartonellosis in Malaysia.* Materials and Methods.* This was a retrospective review of patients treated for ocular bartonellosis in two ophthalmology centers in Malaysia between January 2013 and December 2015. The diagnosis was based on clinical features, supported by a positive* Bartonella* spp. serology.* Results.* Of the 19 patients in our series, females were predominant (63.2%). The mean age was 29.3 years. The majority (63.2%) had unilateral involvement. Five patients (26.3%) had a history of contact with cats. Neuroretinitis was the most common presentation (62.5%). Azithromycin was the antibiotic of choice (42.1%). Concurrent systemic corticosteroids were used in approximately 60% of cases. The presenting visual acuity was worse than 6/18 in approximately 60% of eyes; on final review, 76.9% of eyes had a visual acuity better than 6/18.* Conclusion.* Ocular bartonellosis tends to present with neuroretinitis. Azithromycin is a viable option for treatment. Systemic corticosteroids may be considered in those with poor visual acuity on presentation.

## 1. Introduction

Cat-scratch disease is caused by the Gram-negative, intracellular bacteria* Bartonella henselae*. When ocular involvement is present, the disease is termed “ocular bartonellosis.” The spectrum of ocular bartonellosis manifestation is wide, ranging from neuroretinitis to Parinaud's oculoglandular syndrome, which is an unusual syndrome characterized by granulomas in the eye, preauricular lymphadenopathy, and positive skin test to* Bartonella* spp. antigens [1–4]. In Malaysia, the data on cat-scratch disease, particularly ocular bartonellosis, is limited [5]. We aim to describe the clinical manifestations and visual outcome of ocular bartonellosis in Malaysia. We also outline the relevant investigations for this condition and discuss the management of this zoonotic disease.

## 2. Materials and Methods

We performed a retrospective review of medical records of patients with ocular bartonellosis treated in two ophthalmology centers in Malaysia, between 2013 and 2015. The conduct of the study followed the tenets outlined in the Declaration of Helsinki.

Selection criteria for this study were a diagnosis of ocular bartonellosis based on history of exposure to cats, ocular examination findings, and a single positive serology for* Bartonella* spp. Any two of these were sufficient to make the diagnosis. Patients suspected of ocular bartonellosis were also investigated for other possible infective causes (tuberculosis, syphilis). Serological tests were all performed via immunofluorescence assay (IFA) in the Bacteriology Unit, Institute for Medical Research, Ministry of Health Malaysia. The cut off value was 1 : 12 for Ig M and 1 : 64 for Ig G using a commercial kit to detect* Bartonella henselae* and* Bartonella quintana* (Vircell IFA Bartonella, Paraque Tecnologico, Granada, Spain).

Data collected included onset and duration of visual disturbance, associated systemic symptoms, visual acuity, optic nerve function, anterior and posterior segment examination, treatment regime, and visual outcome. The latter was evaluated based on visual acuity at the last follow-up visit. Visual acuity was assessed monocularly, with the patient seated 6 meters from a Snellen chart. A 6/6 Snellen visual acuity means the patient can read at 6 meters a letter that a normal person would be able to read at 6 meters. The denominator in Snellen visual acuity notation describes the distance at which a normal individual is able to identify a certain symbol, while the numerator refers to the distance required by the patient to identify the same symbol. In this study, a visual acuity of 6/18 or better was considered good, while visual acuity worse than 6/18 was considered poor. Follow-up was until completion of the course of antibiotics, given for a minimum of six weeks.

The data were analyzed using the Statistical Package for Social Science, version 22.0 (SPSS Inc., Chicago, IL, USA). The results are presented as frequencies and percentages.

## 3. Results

A total of 19 patients (26 eyes) with ocular bartonellosis were diagnosed within the study period. The period of follow-up ranged from 3 to 68 weeks, with the mean duration of follow-up being 23.4 weeks. Two patients were lost to follow-up. The duration of symptoms ranged from 5 days to 1 month prior to their first consultation at eye clinic.

The demographic and clinical profile of our subjects is summarized in Table 1. The mean age was 29.3 years. All were Malay, and most of them were female (63.2%, *n* = 12). Only 5 (26.3%) patients gave a history of being scratched or bitten by a cat. The majority (63.2%, *n* = 12) had unilateral involvement.

The most common ocular complaint was blurring of vision, with 61.6% (16 eyes) presenting with a visual acuity worse than 6/18. About one-quarter (7 eyes) had a visual acuity of worse than 6/60. Approximately 62.5% of eyes presented with neuroretinitis. Optic disc edema was seen in 42.1% of patients. One patient presented with features of vasculitis complicated with central retinal vein occlusion, evidenced by macular edema, dilated and tortuous vessels, and flame-shaped hemorrhages. Six patients (31.6%) had signs of uveitis. Ocular findings on presentation and treatment regimes are shown for each patient in Table 2.  Table 3 shows a summary of the ocular presentation and results of serological testing.

Approximately 57.9% of patients had prodromal symptoms, of which headache was the most common. Less common symptoms were fever and upper respiratory tract symptoms.* Bartonella* spp. Ig G serology was positive in all patients, while 68.4% (*n* = 13) had positive Ig M serology. Only a single serology test was performed, due to financial constraints. Hence, disease progression was assessed based on clinical response. The duration of ocular symptoms did not vary between patients with positive Ig G and negative Ig M and those who were positive for both of these.

All patients except two received treatment, which was initiated within the first week of diagnosis. In the case of the latter, this was due to spontaneous recovery before treatment could be initiated. Azithromycin was the most common antimicrobial agent used for treatment (42.1%). Doxycycline, ciprofloxacin, ceftazidime, and cotrimoxazole were other agents used. Approximately 57.9% (*n* = 11) received systemic corticosteroid therapy. Six of these patients were treated with pulsed intravenous corticosteroid therapy, followed by oral corticosteroids. The overall final visual acuity was good, with approximately 76.9% of eyes achieving a good visual outcome (visual acuity of 6/18 or better).

## 4. Discussion

Cat-scratch disease, that is, bartonellosis, is a self-limiting condition in immunocompetent individuals. The spectrum of systemic involvement in bartonellosis ranges from endocarditis to thyroiditis, arthritis, haemolytic anemia, and glomerulonephritis [6–10]. The ocular manifestations of* Bartonella* spp. infection are likewise myriad and have included scotomas [11], papillitis, [12] retinal artery occlusions [13], and even a macular hole [14]. Although systemic cat-scratch disease primarily affects young patients, ocular bartonellosis has a broader age distribution [15]. Our mean age of 29.3 years, with a range of 9 to 58 years, is similar to that of Curi et al. in Brazil [2].

Cats are the main reservoir for* Bartonella* spp., and the main vector of cat-scratch disease is the flea* Ctenocephalides felis* [16]. Cats are common in Malaysia, both as household pets and strays. The prevalence of* Bartonella henselae* in fleas from healthy cats and dogs has been shown to be approximately 33%, while among strays, almost a quarter of them have been found to be seropositive for the bacterium [12, 17]. Exposure to cats or a history of cat-scratch or cat bite is usually present in more than 50% of reported cases of bartonellosis [2, 18–20]. A recent study among healthy blood donors in Brazil revealed that cat contact or a history of tick bite conferred an adjusted odds ratio of* Bartonella* spp. bloodstream infection of 3.4 (95% CI 1.1–9.6) and 3.7 (95% CI 1.3–13.4), respectively [21]. Our study results contradict those, as only approximately one-quarter of our patients had this history. We thus counsel the reader that although a history of cat exposure is common, it is not a prerequisite to make the diagnosis, especially in regions where cats abound [22].

Neuroretinitis in ocular bartonellosis is usually unilateral, but bilateral presentation has been reported [2, 23–25]. In our study, 63.2% of patients presented with unilateral involvement. This is similar to the percentage noted by Kerkhoff et al., who noted that 61.5% of ocular bartonellosis was unilateral [18]. Nearly all (8 out of 9) patients with neuroretinitis had unilateral involvement. Meanwhile, all those with panuveitis (4 patients) had bilateral involvement [18]. Our results differ from those of Curi et al., who reported that 13 out of 24 patients (54.2%) presented with bilateral disease [2].

Neuroretinitis and focal retinochoroiditis are the most common ocular manifestations of cat-scratch disease [2, 19, 20, 23, 24]. In our series, neuroretinitis remained the most common presentation, followed by optic disc swelling. This varies from the findings of Solley et al., who reported that, in his study of 24 patients, the most common finding was discrete retinal infiltrates or choroidal lesions, seen in 83% of patients, followed by optic disc swelling, in 63% of patients [1]. Curi et al. had similar findings to Solley et al., in which the most common ocular manifestation was a small area of retinal infiltrate, which occurred in 11 eyes [1, 2]. In that series, neuroretinitis occurred in only 6 out of 37 eyes (16.2%) [2].

The seroprevalence of antibodies to* Bartonella henselae* varies worldwide, ranging from 3.8% to 50% in various studies [26–28]. To the best of our knowledge, there is no available data on the seroprevalence of* Bartonella henselae* antibodies in our general population. Elevated immunoglobulin M (Ig M) or G (Ig G) for* Bartonella henselae* is suggestive of current or past infection [29]. However, the sensitivity of an Ig M assay using IFA has been found to be lower than that of enzyme immunoassay (EIA) Ig M (50% IFA versus 71.4% EIA) in patients who fulfilled two or more criteria for cat-scratch disease [30]. Despite this, IFA remains the only test of bartonella infection available in our setting. Unfortunately, the IFA used in our bacteriology unit measures antibodies to both* Bartonella henselae* and* Bartonella quintana*; therefore, we were unable to differentiate between these* Bartonella* spp. infections. Polymerase chain reaction (PCR) is emerging as an alternative, highly sensitive method of identifying* Bartonella *spp. in ocular specimens [18, 21]. PCR hybridization assay has shown 86.4% sensitivity in patients who fulfill two or more criteria for cat-scratch disease and 100% sensitivity in patients who fulfill three or more criteria [27]. However, routine PCR is not readily done in our setting due to high cost. We foresee the need for better serological diagnostic tests to guide the diagnosis and treatment of bartonella infection in our practice.

Generally, cat-scratch disease is a self-limiting disease, with good visual prognosis, but treatment may hasten recovery [20, 31]. Early antibiotic treatment may not only speed recovery, but also improve the final visual outcome [20]. According to Bass et al., the mean duration of illness was 2.8 weeks in those with treatment, compared to 14.5 weeks in those without treatment [32]. Rifampicin, gentamicin, cotrimoxazole, ciprofloxacin, and doxycycline have shown efficacy in the treatment of cat-scratch disease [20]. However, doxycycline is contraindicated in children and pregnant women. In mild to moderate disease, the use of oral azithromycin for five-day duration achieves more rapid resolution of lymphadenopathy than conservative management [33]. Azithromycin has become the preferred antimicrobial of choice in treating ocular bartonellosis in our setting due to issues with compliance (daily dosing) and its improved safety profile compared to doxycycline (which is given twice a day and tends to cause gastrointestinal upset). There is no prospective study on the efficacy of azithromycin in treating ocular bartonellosis. However, susceptibility evaluation of* Bartonella henselae* against macrolide antibiotics found that these antibiotics are useful in the treatment of cat-scratch disease [34].

The role of systemic corticosteroids in the treatment of cat-scratch disease is still controversial. Isolated case reports have demonstrated good response to corticosteroids, especially in cases where the presentation is atypical [35–38]. Only one study of ocular bartonellosis has discussed the role of corticosteroids, which gave a good outcome when used in combination with antibiotics, in a series of 14 patients in Japan [19]. Corticosteroids may help control the degree of intraocular inflammation and optic neuropathy due to cat-scratch disease. In our study, systemic corticosteroid therapy was used in patients who had significantly decreased visual acuity on presentation. However, we do not believe that topical corticosteroids play a significant role in the management of this condition, as their penetration to the posterior segment of the eye is limited.

Our study has several limitations. First, its retrospective nature limits the amount of information available. Secondly, although our data was collected from two institutions, the demographic and clinical features of this disease may show a wider global variation than currently reported, due to the different socioeconomic and bacteriology profile in different regions. Direct comparison with previous literature is also not possible due to the relatively small numbers of patients in most studies, as well as differences in study design, including inclusion criteria and type of serological assay used. Larger scale studies and standardized diagnostic criteria are required to consolidate our findings.

## 5. Conclusion

Ocular bartonellosis tends to present with blurring of vision, which is associated with prodromal symptoms in more than half of these cases. The most common ocular signs are neuroretinitis, papillitis, and uveitis. Azithromycin is a viable option for treatment. Systemic corticosteroids may be considered in those with poor visual acuity on presentation.

## Figures and Tables

**Table 1:** Patient demographic profile.

Demographic data	*n* (%)
Age in year (mean, SD)	29.3, 13.2

Race	
Malay	19 (100.0)

Sex	
Male	7 (36.8)
Female	12 (63.2)

Cat exposure	
No	14 (73.7)
Yes (contact or scratch)	5 (26.3)

Systemic symptoms	
No	8 (42.1)
Yes	11 (57.9)
Headache	7 (43.8)
Upper respiratory tract infection	2 (12.5)
Fever	6 (37.5)
Lymphadenopathy	1 (6.2)

**Table 2:** Ocular Presentation of Bartonellosis and Treatment Regime.

Case number	Lat	Initial VA	Final VA	Positive serology (titre)	Anterior uveitis	Neuroretinitis	Optic disc edema	Others	Rx	Corticosteroid therapy
(1)	B	RE 6/18 LE 6/24	RE 6/7.5 LE 6/7.5	Ig M (1 : 96) /Ig G (1 : 56)		Yes			Azi	Oral
(2)	R	6/120	6/21	Ig M (1 : 24) /Ig G (1 : 128)	Yes	Yes			Azi	IV then oral
(3)	R	6/24	6/6	Ig M (n/a) /Ig G (n/a)			Yes	Focal choroiditis	Azi	None
(4)	L	6/120	6/45	Ig M (1 : 24) /Ig G (1 : 128)			Yes	Retinal infiltrates	Azi	Oral
(5)	L	6/7.5	6/6	Ig M (1 : 48) /Ig G (1 : 256)			Yes		Azi	None
(6)	L	2/60	6/6	Ig M/(1 : 24) /Ig G (1 : 128)	Yes	Yes		CWS	Azi	Topical
(7)	B	RE 6/30 LE 6/21	RE 6/9 LE 6/6	Ig M/(1 : 48) /Ig G (1 : 128)		Yes			Cip	IV only
(8)	B	RE 6/7.5 LE 6/12	RE 6/7.5 LE 6/7.5	Ig G (1 : 128)		Yes			Dox	None
(9)	L	6/24	LE 6/6	Ig M (1 : 24) /Ig G (1 : 128)			Yes		Dox	Topical
(10)	B	RE 6/6 LE 6/6	RE 6/6 LE 6/6	Ig M (1 : 24) /Ig G (1 : 128)	Yes		Yes		None	None
(11)	B	RE 6/6 LE 2/60	RE 6/6 LE 6/9	Ig M (1 : 96) /Ig G (1 : 512)		Yes		Retinal infiltrates	Azi	Oral
(12)	B	RE CF LE 6/36	RE 6/9 LE 6/6	Ig G (1 : 128)		Yes		CWS/retinal hemorrhage	Ceft	None
(13)	B	RE 6/9 LE 6/60	RE 6/9 LE 6/24	Ig G (1 : 128)			Yes		Ceft/Azi	IV then oral
(14)	L	1/60	6/24	Ig M (1 : 24) /Ig G (1 : 128)		Yes			Cefu	Oral
(15)	R	HM	HM	Ig M (1 : 24) /Ig G (1 : 128)			Yes	Macular edema/ vasculitis/dilated vessels/retinal hemorrhage	Ceft/Dox	IV then oral
(16)	L	6/60	6/36	Ig M (1 : 24) /Ig G (1 : 128)		Yes			Dox	IV then oral
(17)	L	6/18	6/9	Ig G (1 : 128)			Yes	Vasculitis/ dilated vessels/ perivascular sheathing	Co-t	Oral
(18)	R	6/9	6/6	Ig G (1 : 128)	Yes				Ceft/Cefu	Topical
(19)	L	6/24	6/6	Ig G (1 : 128)	Yes	Yes		Retinal hemorrhage	None	IV then oral

Lat, laterality; R, right; L, left; B, bilateral; VA: visual acuity; CF, counting finger; HM, hand movement; Ig, immunoglobulin; Rx, antibiotic; Azi, azithromycin; Cip, ciprofloxacin; Dox, doxycycline; Ceft, ceftazidime; Cefu, cefuroxime; Co-t, cotrimoxazole; IV, intravenous.

**Table 3:** Presenting features of ocular bartonellosis.

Parameters	*n* (%)
Laterality	
Left	8 (42.1)
Right	4 (21.1)
Bilateral	7 (36.8)

Ocular presentation	
Anterior uveitis	6 (31.6)
Neuroretinitis	10 (62.5)
Optic disc edema	8 (42.1)
Focal choroiditis	1 (5.3)
Retinal infiltrates	2 (10.5)
Cotton wool spots	1 (5.3)
Retinal hemorrhages	3 (15.8)
Macular edema	1 (5.3)
Vasculitis	2 (10.5)

IFA serology positivity	
Ig M	13 (68.4)
Ig G	19 (100.0)

IFA, immunofluorescent assay; Ig, immunoglobulin.
